# Dynamics of a three-dimensional oil drop driven by a surface acoustic wave over topography

**DOI:** 10.1140/epje/s10189-026-00604-9

**Published:** 2026-06-29

**Authors:** Mark Fasano, Yifan Li, Javier A. Diez, Ofer Manor, Linda J. Cummings, Lou Kondic

**Affiliations:** 1 Department of Mathematical Sciences and Center for Applied Mathematics and Statistics, New Jersey Institute of Technology, Newark, NJ 07102 USA; 2 Department of Chemical Engineering, Technion - Israel Institute of Technology, 32000 Haifa, Israel; 3 Instituto de Física Arroyo Seco, Universidad Nacional del Centro de la Provincia de Buenos Aires and CIFICEN-CONICET-CICPBA, 7000 Tandil, Argentina

## Abstract

**Abstract:**

We perform three-dimensional simulations of SAW-driven spreading of silicone oil drops on flat substrates and over solid obstacles. The resulting model takes the form of a three-dimensional long-wave thin-film equation incorporating capillary, gravitational, and SAW-induced acoustic stresses. A key feature of the formulation is a smooth attenuation function that localizes acoustic forcing within the bulk drop while avoiding spurious transverse discontinuities. Comparisons with results of earlier 2D formulations demonstrate qualitatively similar dynamics, albeit the additional spatial dimension permits transverse mass redistribution driven by capillarity, which leads to slower streamwise spreading and slightly lower drop apexes than predicted by 2D models. The model is applied to SAW induced dynamic wetting of flat substrates and solid obstacles and is representative of experimental geometries. Quantitative comparisons with experimental observations show good agreement for front propagation and obstacle climbing dynamics. In particular, improved agreement with the experimentally observed dependence of the liquid climbing time over obstacles on SAW amplitude is obtained when the fully three-dimensional formulation is used.

**Graphical abstract:**

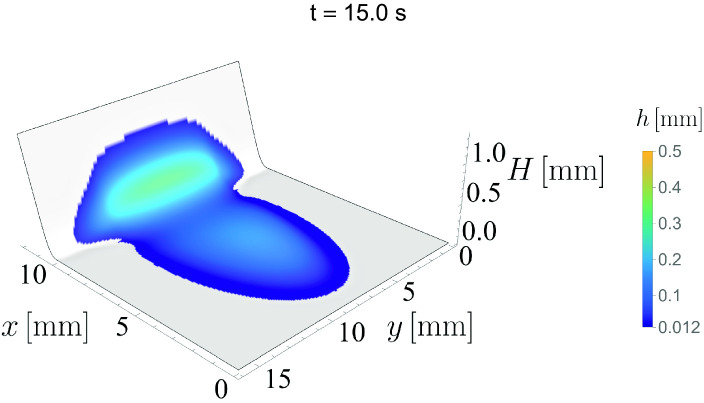

**Supplementary Information:**

The online version contains supplementary material available at 10.1140/epje/s10189-026-00604-9.

## Introduction

Dynamic wetting and thin-film spreading on solid substrates are central to a wide range of natural and technological processes. In the context of adaptive, flexible, and switchable substrates, external actuation provides a means of modifying wetting dynamics without changing the liquid itself. One such mechanism is substrate vibration, which can induce drop deformation, translation, depinning, and coating flows. In this sense, surface acoustic wave (SAW)-driven spreading represents a high-frequency realization of switchable wetting, where the effective substrate forcing is controlled through the applied acoustic signal.

Classical long-wave theory has long provided a reduced-order framework for describing gravity- and capillarity-driven flows, with comprehensive treatments given by Oron et al. [1] and Craster and Matar [2]. In addition to these canonical driving mechanisms, mechanical vibrations of a solid substrate can induce steady flow in an adjacent liquid through acoustic streaming. In particular, megahertz-frequency Rayleigh surface acoustic waves (SAWs) have emerged as a powerful means of actuating droplets and films in microfluidic systems. Related studies have shown that low-frequency substrate vibrations can also drive drop motion and uphill climbing on inclined substrates [3, 4]. Although these works concern vibration-induced wetting rather than SAW actuation, they illustrate the broader principle that substrate motion can be used to control drop mobility.

The study of steady flow by high-frequency oscillatory motion traces back to the classical works of Rayleigh [5] and Nyborg [6], later formalized in the acoustic streaming theory of Lighthill [7] and revisited in modern treatments such as Vanneste and Bühler [8]. When a Rayleigh SAW propagates along a solid substrate in contact with a viscous liquid, part of its energy leaks into the fluid, generating an attenuated acoustic field. Quadratic nonlinear interactions of this oscillatory field produce a steady bulk body force, often referred to as Eckart streaming, while viscous boundary layers near the solid surface give rise to Rayleigh streaming. These mechanisms have been observed and characterized experimentally in a wide range of acoustofluidic systems [9–12].

SAW-induced transport has been exploited in applications ranging from microfluidic actuation [13–15] to thin-film coating and wetting phenomena [16–19]. In such systems, droplets subjected to SAW excitation undergo rapid deformation, translation, and spreading, often leaving behind thin residual films. Despite the extensive experimental literature, predictive reduced-order theoretical descriptions that consistently couple acoustic streaming with capillarity and gravity have only recently been developed.

Fasano et al. [20] derived a long-wave-type model for SAW-driven spreading on flat substrates, incorporating exponential attenuation of the acoustic field beneath the liquid bulk. That work demonstrates that the steady translation speed of a millimeter-scale drop arises from a balance between capillary reshaping and a nonconservative acoustic flux generated by streaming. The model captures the experimentally observed dependence of front speed on SAW amplitude and clarifies the role of attenuation length in selecting the drop’s quasi-steady traveling-wave profile. Subsequently, Li et al. [21] extended this framework to account for substrate topography, showing how obstacle geometry modifies attenuation and leads to distinct coating regimes. Both of these studies, however, are restricted to two-dimensional (2D) geometries assuming translational invariance in the transverse direction. In experiments, SAW-driven droplets are inherently three-dimensional (3D): the footprint evolves in both streamwise and lateral directions, and attenuation varies across the full drop footprint. Extending the model to three dimensions is not a trivial geometric generalization. In the 2D formulation, acoustic attenuation is applied only within the bulk of the drop by shifting the attenuation profile downstream of a cutoff thickness, which represents the transition between the bulk drop and the thin prewetted layer surrounding it. This construction ensures that acoustic forcing acts only within the bulk drop and not in the surrounding film. When extended directly to three dimensions, however, this sharp switching produces discontinuities in acoustic forcing across the drop footprint, generating artificial pressure gradients and unphysical behavior. A consistent 3D formulation must therefore localize attenuation within the bulk while maintaining smoothness in both streamwise and transverse directions.

The objective of the present work is to construct and analyze a 3D long-wave model for SAW-driven droplet dynamics that resolves this attenuation modeling challenge. Building on the theoretical framework developed by Fasano et al. [20] and Li et al. [21], we introduce a smooth streamwise attenuation factor coupled to a modified Helmholtz equation that eliminates spurious transverse forcing while preserving the physical exponential decay of the acoustic field. The resulting model predicts dynamical features absent in 2D, including a gradual streamwise elongation of the drop footprint arising from subtle differences in acoustic forcing near the rear of the drop in the 2D and 3D formulations, discussed in detail in Sect. 3.2. We compare the predictions of the 3D model with experimental measurements and with those of the earlier 2D formulation [20]. We find that, while long-time front speeds remain comparable, the 3D drop dynamics exhibit distinct transient behavior and systematic growth in streamwise extent. By resolving the attenuation structure in 3D, the present work provides a consistent thin-film framework for describing SAW-driven droplet motion in realistic geometries.

The remainder of the paper is structured as follows. In Sect. 2, we briefly present experimental results that motivated this study. Then in Sect. 3, we formulate the 3D long-wave model: Sect. 3.1 presents the governing equations, Sect. 3.2 details the construction of the acoustic attenuation function, and Sect. 3.3 introduces the nondimensionalization employed. We present numerical results in Sect. 4, including comparison with experimental measurements and the corresponding 2D formulation. Finally, Sect. 5 summarizes the main findings and discusses their implications. Technical details concerning the numerical implementation of the model described in Sect. 3 are provided in Appendix A.  

## Experiments

The experiments that motivated the present 3D model were previously reported in detail by Fasano et al. [20] and Li et al. [21]. Here we provide only a brief description of the experimental configuration and a representative example illustrating the physical phenomena of interest. A comparison of selected experimental measurements with model simulations is provided in Sect. 4 later (Fig. 6).

**Fig. 1:**
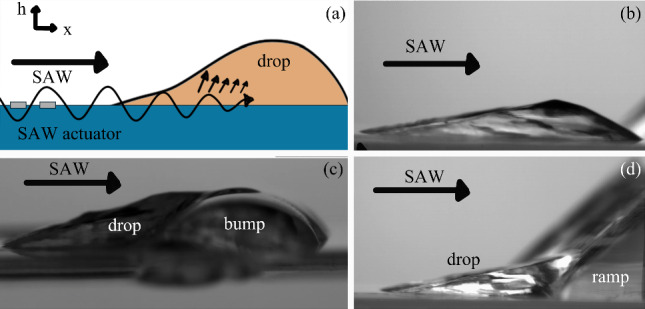
Sketch (**a**) and snapshots (**b**–**d**) from a typical experiment. (**a**) A surface acoustic wave (SAW) propagates in the solid substrate (blue); when the SAW encounters an oil film or an oil drop, power leaks into the liquid (upward arrow). (**b**–**d**) Snapshots of the drop driven forward induced by the SAW: (**b**) moving freely, (**c**) moving over a bump, and (**d**) climbing a ramp

A millimeter-scale thick silicone oil film (kinematic viscosity range of $\nu =50-500$ cSt, oil volume range of $V_d=3-8$ mm$^3$) is placed on a transducer that generates a propagating surface acoustic wave – a Rayleigh wave – confined to a depth below the solid surface of the transducer of approximately two wavelengths. The acoustic contribution to oil transport is characterized by the normal displacement amplitude of the substrate surface by the SAW, denoted $A_n$. The experimentally reported SAW amplitude, $A_n$, corresponds to the maximum normal displacement of the lithium niobate substrate surface generated by the propagating Rayleigh SAW. The substrate displacement is measured using scanning laser Doppler vibrometry (LDV), which provides the relation between applied voltage and SAW amplitude [20, 21]. The SAW power is transferred to the oil film when the SAW in the solid substrate of the transducer comes into contact with the oil film placed atop the transducer, as shown in Fig. 1(a) (the wavy arrow represents the SAW). The SAW diffracts a same-frequency ultrasound which generates a body force in the oil film, resulting in bulk streaming, i.e., Eckart streaming. The oil film deforms and spreads along the path of the SAW, as shown in Fig. 1(b), leaving a residual wetting oil film behind. In addition to spreading on a flat substrate [20], experiments were performed on substrates with prescribed solid topography fabricated from cured elastomer, Polydimethylsiloxane (PDMS)  [21]. In the present work, we simulate experimentalconfigurations in which the advancing oil film encounters localized (bump, Fig. 1c) or inclined (ramp, Fig. 1d) obstacles.

Figure 1(b–d) illustrate the various cases of SAW-driven spreading. For the obstacle experiments in Fig. 1(c, d): as the drop approaches the obstacle, acoustic forcing drives the front contact line upward along the incline. The interplay between capillarity, gravity, and SAW-induced forcing determines whether the drop arrests at a finite height or continues to climb. For sufficiently strong forcing the drop fully coats the obstacle and continues advancing beyond it; for weaker forcing, the front becomes arrested. During the translation and climbing, lateral spreading also occurs, playing a role not captured by the 2D model  [21] and motivating the development of the present 3D model and simulations.

The experiments presented here serve solely to illustrate the qualitative behavior that the present 3D model seeks to capture. In what follows, we focus on the formulation and analysis of a fully 3D long-wave model capable of resolving the transverse structure and attenuation effects that arise in such configurations.

## Model

We develop a 3D long-wave model for a viscous oil drop driven by a Rayleigh-type SAW propagating in the underlying substrate. The formulation extends the earlier 2D frameworks [20, 21], which themselves build upon classical treatments of leaky SAW-induced streaming [8, 22]. Since the leading-order acoustics and streaming derivation are unchanged from those works, we briefly summarize the key results and focus on the extension of the acoustic attenuation function to a fully 3D geometry.

### Governing equations

Following [20, 21], we assume a small Mach number and decompose the velocity, pressure, and density fields into leading-order quiescent fields, oscillatory first-order components, and time-averaged second-order components. The first-order flow corresponds to an irrotational acoustic field induced by the leaky SAW and satisfies a damped Helmholtz equation with boundary conditions determined by classical elastic wave theory in solids [23]. The resulting acoustic velocity field decays exponentially both along the direction of propagation (*x*-axis) and normal to the substrate (*z*-axis). Its sole role in the present model is to generate, through quadratic nonlinear interactions, a non-zero time-averaged Reynolds stress that drives steady streaming.

Time-averaging over the acoustic period yields a second-order Stokes problem forced by the divergence of the Reynolds stress. As shown in detail in [20, 21], this produces a body force, $\textbf{F}_s$, with magnitude of the form

1 $$\begin{aligned} |\textbf{F}_s| \sim P_0e^{-2(k_\textrm{s,i}x+K_zz)}=P_0\, \Psi (x,z), \end{aligned}$$

where the attenuation coefficients ($k_\textrm{s,i},K_z$) depend on material and wave properties and $P_0=\rho _0A^2\omega ^2$ scales acoustic (Reynolds) stress generated by the SAW (here, $\rho _0$ is the constant, leading order term in the asymptotic expansion of the oil film density in the small acoustic Mach number, $\omega$ is the angular frequency of the SAW, and *A* is the SAW normal displacement amplitude at the solid surface in the computational domain (to differentiate from the same property in the experiment, which we term $A_n$). Under a long-wave approximation, integrating across the film thickness and enforcing a Laplace pressure boundary condition at the free surface yields an evolution equation for the film height, *h*(*x*, *y*, *t*), over a substrate with a topographical obstacle, *s*(*x*), of the form

2 $$\begin{aligned} \partial _t h+\nabla _\parallel \cdot \textbf{Q}=0, \qquad \nabla _{\parallel }=\begin{pmatrix} \partial _x \\ \partial _y \end{pmatrix} \end{aligned}$$

where the flux, $\textbf{Q}$, consists of both a conservative ($\textbf{Q}_\textrm{c}$, with capillary, gravitational, and acoustic components) and a nonconservative ($\textbf{Q}_\textrm{nc}$, solely acoustic) contribution ($\textbf{Q}=\textbf{Q}_\textrm{c}+\textbf{Q}_\textrm{nc}$). The flux contributions are of the form

3 $$\begin{aligned} \textbf{Q}_\textrm{c}&= \begin{pmatrix} Q_{\textrm{c},x}\\ Q_{\textrm{c},y} \end{pmatrix} = -\frac{h^3}{3\mu }\nabla _{\parallel } P, \end{aligned}$$

4 $$\begin{aligned} Q_{\textrm{nc},x}&= -\mathcal {C}\frac{P_0}{8\mu K_z^4} \,\Psi (x,h+s) \nonumber \\&\quad \times \left( 2K_z^2h^2 - 1 + e^{2K_zh}(1-2K_zh) \right) , \end{aligned}$$

5 $$\begin{aligned} Q_{\textrm{nc},y}&= 0, \end{aligned}$$

with effective pressure

6 $$\begin{aligned} \mathcal {P}=\rho _0g\Big (h+s\Big )-\gamma \Big (\mathcal {K}+\mathcal {K}_s\Big )+\frac{C_zP_0}{2K_z}\Psi (x,h+s). \end{aligned}$$

In the model, $\mu$ is the dynamic oil viscosity, *g* is the acceleration due to gravity, $\mathcal {K}$ and $\mathcal {K}_s$ are the mean curvatures of the oil and of the topographical obstacle, respectively (both under the long-wave approximation). Furthermore, $C_z$ is a prefactor of the *z*-component of the acoustic force $\textbf{F}_s$, $\Psi (x,h+s)$ is an acoustic attenuation function, and the parameter $\mathcal {C}=K_zC_x-k_\textrm{s,i}C_z$ measures the nonconservative part of the acoustic force. In earlier 2D formulations [20, 21] we used a different attenuation function ($\psi$); however, here we slightly modify the definition to account for transverse variations smoothly. The complete expressions follow directly from [20, 21] and are not reproduced here for brevity. Specifically, we point the interested reader to Section 3.1 and Appendix A of [20] for a detailed discussion of the acoustic parameters that arise in the model and their numerical values.

### Modeling acoustic attenuation

The principal modeling challenge in extending the 2D formulation [21] to 3D lies in constructing an attenuation function that remains consistent across a fully 2D drop footprint, which is connected with a surrounding prewetted layer. In experiments, the SAW is appreciably attenuated only within the bulk of the drop and obstacle. In the 2D model developed earlier [21], attenuation is activated only where $h+s>h^*$ (where $h^*$ is a cutoff thickness below which fluid transport is governed by other mechanisms), with a sharp downstream shift that eliminates acoustic forcing in the prewetted region behind the drop.

In 3D, the corresponding cutoff location becomes a spanwise-dependent level-set curve. A direct extension of the 2D sharp switching introduces discontinuities in the transverse, *y*-direction, producing spurious transverse pressure gradients and numerical artifacts in the prewetted regions. To avoid this, we introduce two coupled smoothing mechanisms. First, a streamwise attenuation factor $\psi ^x$ which satisfies

7 $$\begin{aligned} \partial _x\psi ^x+2k_\textrm{s,i}H_{\delta }\Big ((h{+}s)-h^*\Big )\psi ^x=0, \qquad \psi ^x\bigg |_{x=0}=1, \nonumber \\ \end{aligned}$$

where $H_\delta$ is a smooth approximation of the Heaviside function that transitions from 0 to 1 across a narrow region of thickness $O(\delta )$ around $h+s=h^*$, ensuring a continuous activation of acoustic forcing within the bulk of the film. This produces an attenuation function identical to that used in the 2D formulation and thus still inherently leads to spurious transverse pressure gradients in the prewetted layer. To regularize this transition and maintain smooth forcing across the footprint, we introduce a further smoothing field *G*(*x*, *y*, *t*) defined by the Helmholtz-type equation

8 $$\begin{aligned} -\delta _G^2 \nabla _\parallel ^2G+ G = H_{\delta }\Big ((h+s)-h^*\Big ), \end{aligned}$$

with no-flux boundary conditions $G_x=0=G_y$ applied at the edges of the computational domain. Here, $\delta _G$ sets the characteristic length scale over which the transition between attenuated and non-attenuated regions is smoothed. Since this smoothing is introduced purely for numerical regularization, $\delta _G$ does not correspond to a physical parameter. In practice, its value is selected through a convergence study: starting from a value that yields stable and computationally efficient simulations, $\delta _G$ is systematically reduced until further decreases produce only negligible changes in the solution while maintaining numerical stability. The effective attenuation factor entering the pressure and nonconservative flux is then

9 $$\begin{aligned} \Psi =G\,\psi ^x\exp (-2K_z(h+s-h^*)), \end{aligned}$$

where *G* is the solution of Eq. 8. This construction preserves the intended bulk attenuation while maintaining smoothness in both streamwise and transverse directions. In all numerical simulations of Eqs. (2)–(6), we use Eq. (9) to model $\Psi$. The dynamical implications of this modification are discussed in Sect. 4.

### Nondimensionalization

The problem is nondimensionalized using a single length scale $\ell$, pressure scale $\gamma /\ell$, and time scale $3\mu \ell /\gamma$; all acoustic attenuation factors ($k_\textrm{s,i},K_z$) and acoustic forcing prefactors ($C_x,C_z$) are scaled by $\ell$ while $\mathcal {C}$ is scaled by $\ell ^2$. The nondimensional governing equation is then of the form

10 $$\begin{aligned} \partial _t h&+ \nabla _\parallel \cdot \Big ( -h^3 \nabla _\parallel P + \textbf{Q}_\textrm{nc} \Big ) = 0, \end{aligned}$$

11 $$\begin{aligned} Q_{\textrm{nc},x}&= -\mathcal {C}\,\frac{3S}{8K_z^4}\,\Psi (x,h+s) \nonumber \\&\quad \times \left( 2K_z^2h^2 - 1 + e^{2K_zh}(1-2K_zh) \right) , \end{aligned}$$

12 $$\begin{aligned} Q_{\textrm{nc},y}&= 0, \end{aligned}$$

with

13 $$\begin{aligned} \mathcal {P}=-&\Big (\mathcal {K}+\mathcal {K}_s\Big )+\text {Bo}\, \Big (h+s\Big ) + \frac{\mathcal {S}C_z}{2K_z}\Psi (x,h+s), \end{aligned}$$

14 $$\begin{aligned}&\text {Bo}=\frac{\rho _0g\ell ^2}{\gamma }, \qquad \mathcal {S}=\frac{P_0\ell }{\gamma }, \end{aligned}$$

where $\text {Bo}$ is the Bond number measuring the ratio of gravitational to capillary forces, $\Psi$ as defined by Eq. (9), and $\mathcal {S}$ is a nondimensional parameter measuring the relative strengths of the SAW-induced acoustic stress and surface tension. The parameter values used in the simulations are summarized in Table 1.

**Table 1:** Dimensional, nondimensional, and numerical parameters used in simulations. Unless otherwise stated, all parameters are held fixed; only the forcing parameter *S* varies with acoustic amplitude *A*

Dimensional parameters	Nondimensional parameters
$\nu = 50~\textrm{cSt}$	$Bo = 0.45$
$\gamma = 20.8~\mathrm {dyn/cm}$	$K_z = 0.28$
$\rho _0 = 0.96~\mathrm {g/cm^3}$	$k_{s,i} = 0.37$
$g = 9.8~\mathrm {m/s^2}$	$C_x = 0.15$
$\omega = 125.7~\textrm{MHz}$	$C_z = 0.42$
$A = 0.8{-}6~\textrm{nm}$	$\mathcal {C} = -0.11$
$A_n = 0.64{-}1.92~\textrm{nm}$	$S = 0.47{-}26.24$
$$\ell = 1~\textrm{mm}$$	
Numerical parameters	
$$h_* = 0.017$$	
$$h_p = 0.01$$	
$$h_d = 0.497$$	
$$r_d = 3.2$$	
$$\delta _G = 5\times 10^{-5}$$	
$$\mathcal {V}_\textrm{d}=8$$	

## Numerical results

In this section, we discuss the predictions of the model developed in Sect. 3 and compare them with both experimental observations and the previously developed 2D formulation of Fasano et al. [20]. Particular emphasis is placed on identifying dynamical differences that arise from the additional spatial dimension and from the modified acoustic attenuation construction described in Sect. 3.2. In our simulations, we solve Eq. (10) coupled to Eqs. (7) and (8) with effective pressure defined by Eq. (13). As the initial condition, we consider a 3D parabolic drop of the form

15 $$\begin{aligned} h(x,y,0)&=h_\textrm{p}+ \bigg (h_\textrm{d} - h_\textrm{p}\bigg ) \nonumber \\&\quad \times \max \left( 1 - \frac{(x - x_\textrm{d})^2 + (y - y_\textrm{d})^2}{r_\textrm{d}^{\,2}},\,0\right) , \end{aligned}$$

where $h_\textrm{p}=0.01$ is the prewetted film thickness, $h_\textrm{d}=(2\mathcal {V}_\textrm{d})/(\pi r_\textrm{d}^2)$ is the maximum height of the drop, $(x_\textrm{d},y_\textrm{d})$ is the center of the drop, and $r_\textrm{d}=3.2$ is the initial drop radius. At all domain boundaries, we impose $h=h_\textrm{p}$ with a zero-normal-flux condition, so that the prewetted layer thickness is maintained at the boundary and no net mass crosses the boundary. The computational domain $x\in [0,W_x]$ and $y\in [0,W_y]$ was chosen on a case-by-case basis, with $W_x$ and $W_y$ sufficiently large that the evolving film did not interact with the boundaries over the full simulated time interval. Across the simulations reported here $W_x$ ranged from 16 to 20 while $W_y=16$ in all cases.

**Fig. 2:**
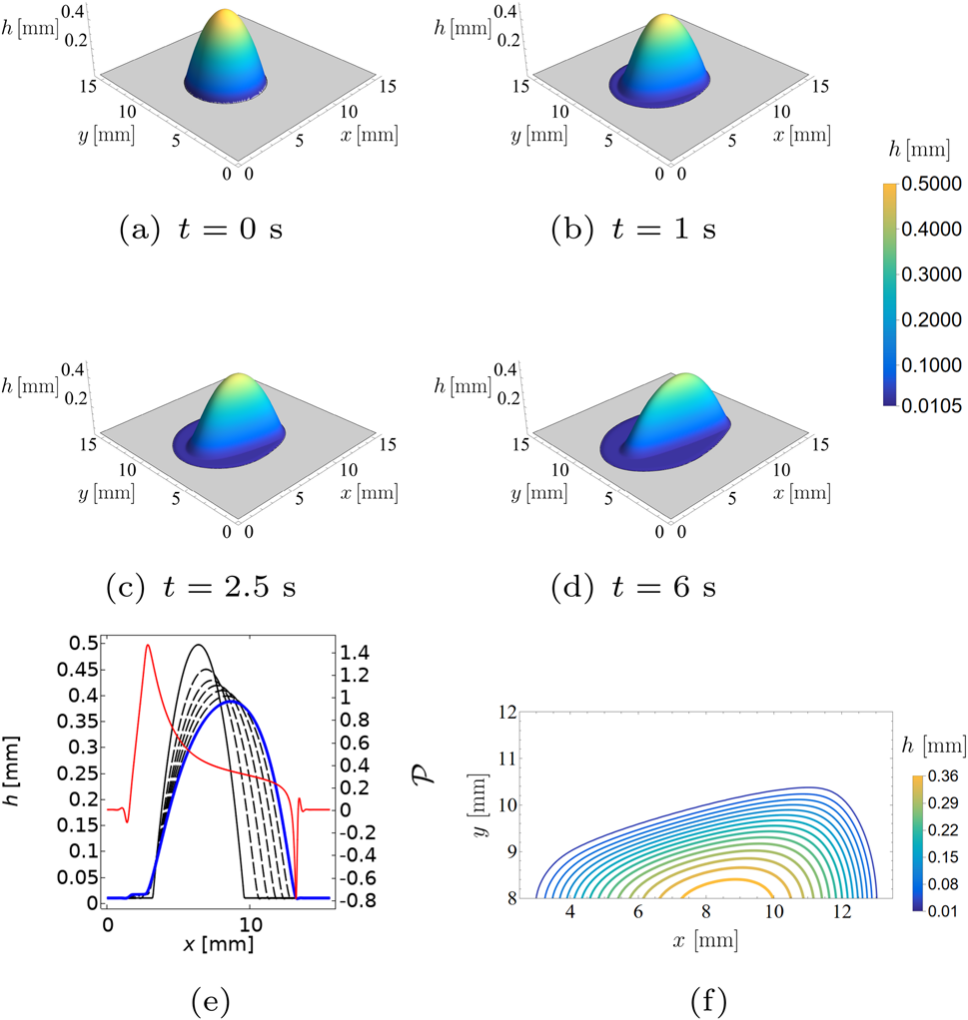
(**a**)–(**d**) Evolution of thickness profile, *h*(*x*, *y*, *t*), for $A=2$ nm on a flat substrate ($s=0$). (**e**) Snapshots of the thickness profile along the midline $y=W_y/2$; the black solid line corresponds to the initial condition, the dashed black lines to the profiles at output time steps $\Delta t=1$ s, and the solid blue line to $t=6$ s. The red line shows the effective pressure, $\mathcal {P}$, at $t=6$ s. (**f**) Contour plot of the drop thickness at $t=6$ s

### Spreading on a flat substrate

We first consider spreading on a flat substrate ($s=0$) for an oil drop of volume $V_\textrm{d} =8$ mm$^3$ subjected to SAW forcing of amplitude $A=2$ nm. Figure 2(a)–(d) shows snapshots of the drop thickness *h*(*x*, *y*, *t*) as the drop translates in the direction of wave propagation (which is always $+x$). In these plots, the prewetted layer is shaded gray while the bulk thickness is represented using the accompanying colorbar. Specifically, grid points satisfying $h\le h_\textrm{p}+\epsilon$ are rendered in gray. In the flat-substrate simulations, we take $\epsilon =0.005$, while for the obstacle cases ($s\ne 0$) a slightly larger value $\epsilon =0.02$ is used to ensure a clear separation between the prewetted layer and the bulk film. This allows the thin residual film left behind the advancing drop (of thickness $h_\textrm{p}\lesssim h\lesssim h^*$) to be clearly distinguished from the thicker bulk region (see Fig. 2b–d). The thickness of this residual film depends on the value of $h^*$ and represents the fluid thickness below which the mechanism of Eckart streaming becomes weak and mass transport is dominated by capillary contributions and other weak acoustic effects (e.g. Rayleigh streaming and acoustic radiation pressure), which are shadowed by Eckart streaming when the film is thicker. The spreading process separates the fluid into three distinct regions: the prewetted layer (shown in gray), the thin residual film left behind by the advancing drop (dark blue puddle), and the bulk drop itself. As the drop advances, fluid is continuously deposited into the residual film along the sides of the footprint, which evolves anisotropically as the drop advances. Its extent in the streamwise *x*-direction increases while the solution remains symmetric with respect to the symmetry line $y=W_y/2$. The streamwise growth of the footprint is a distinctive feature of the 3D simulations and results from the modified modeling of the attenuation function $\Psi$ in the present formulation compared with the attenuation function $\psi$ used in the earlier 2D model [20].

Figure 2(e) shows cross-sections of the thickness profile along $y=W_y/2$ at successive times. Comparing the solid black curve (initial condition) to the solid blue curve (solution at $t=6$ s), one can clearly see an increase in width in the streamwise direction. In both the 2D and the present 3D formulation, the acoustic forcing vanishes in the prewetted layer since the attenuation function is spatially constant. However, in the 2D model the transition from the bulk to the prewetted layer is effectively sharp, so no forcing is generated near the rear of the drop. In contrast, the 3D attenuation is constructed to vary smoothly, which introduces a small but finite gradient in this transition region and thus a weak additional forcing near the rear. This effect is reflected in the pressure profile shown. Figure 2(f) shows a contour plot of the drop above the symmetry line $y=W_y/2$. The contours illustrate the anisotropic spreading of the drop footprint: while the residual film extends laterally in the *y*-direction, the bulk drop remains concentrated near the symmetry line and elongates in the streamwise *x*-direction.

**Fig. 3:**
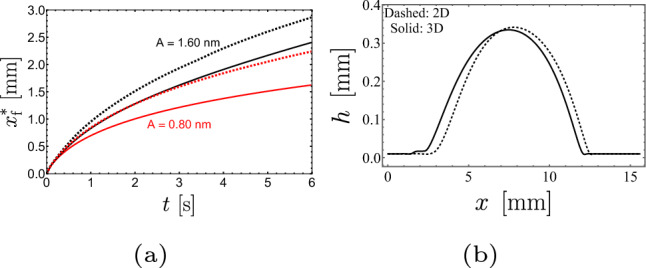
(**a**) Comparison of front position of drops on flat substrates for 2D (dashed lines) and 3D models (solid lines). (**b**) Height profiles for $A=1.6$ nm at $t=6$ s where the dashed curve denotes the 2D model and the solid curve the 3D model

To quantify the influence of spatial dimensionality (2D versus 3D), we compare in Fig. 3(a) the front position predicted by the present 3D model with the results obtained from the 2D formulation [20]. The front location $x_\textrm{f}^*(t)$ is defined as the rightmost point at which $h=h^*$; in practice, for the 3D model, this location is determined numerically along the line of symmetry $y=W_y/2$ by identifying the furthest downstream crossing of the level set. The two models predict qualitatively similar behavior, but quantitative differences arise during the early transient stage. During this period, the initially symmetric drop (circular in footprint) deforms into an asymmetric shape as it translates under acoustic forcing. In particular, the 3D model predicts a slightly slower advance of the front during the early stages of spreading. At longer times the propagation speeds predicted by the two models become comparable. We attribute this difference to the fact that in the 3D geometry fluid is allowed to redistribute laterally in the *y*-direction. Capillary forces therefore promote transverse spreading of the drop, which reduces the amount of fluid transported in the streamwise direction and thereby slows the front’s advance. Additionally, in the 3D formulation, the small but finite gradient in the acoustic attenuation function in the transition region near the rear of the drop introduced by the smooth attenuation profile, produces a weak opposing pressure gradient that reduces the local acoustic forcing and slows the rear relative to the front. As a result, fluid is transported forward while the rear region advances more slowly (relative to the 2D formulation), producing a modest streamwise elongation of the drop footprint compared with the 2D model. These effects are further illustrated in Fig. 3(b), which compares height profiles from the two models at $t=6$ s (where for the 3D model we plot the solution along the line of symmetry $y=W_y/2$). The 3D solution exhibits a slightly lower apex height, which reflects the redistribution of mass in the *y*-direction, and a slightly wider profile.

### Spreading over a ramp obstacle

**Fig. 4:**
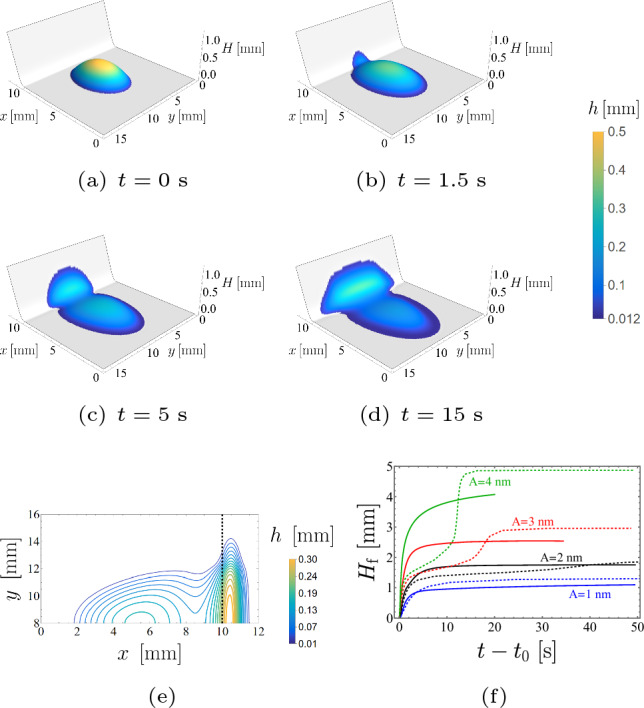
(**a**)–(**d**) Snapshots of the surface, $H=h+s_\textrm{r}$, plotted at specified times with the colorbar indicating film thickness, *h*, so small variations in film thickness are easily identifiable. (**e**) Contour plot of film thickness, *h*, for $A=1$ nm at $t=15$ s; the dashed black line denotes the start of the ramp obstacle. (**f**) Climbing height, $H_\textrm{f}$, as a function of time for oil drops on a ramp where the dashed curves indicate the 2D results and solid curves the 3D results

We next investigate spreading over a ramp-shaped obstacle, designed to approximate the experimental geometry of Fig. 1(d). The ramp is modeled by the profile:

16 $$\begin{aligned} s_\textrm{r}(x)= \left\{ \begin{array}{lll} 0, & \quad x <x_\textrm{o}, \\ \frac{h_\textrm{o}}{w_\textrm{o}}(x-x_\textrm{o}), & \quad x > x_\textrm{o}, \end{array} \right. \end{aligned}$$

with a smoothing region near $x=x_\textrm{o}$ of width 0.5 to ensure continuous first and second derivatives; we use the parameters $h_\textrm{o}=3.2,\, w_\textrm{o}=3.5$, and $x_\textrm{o}=10.5$. Figure 4(a)–(d) shows snapshots of the evolving surface $H=h+s_\textrm{r}$ at several times during the simulation for SAW amplitude $A=1$ nm. In these plots, the vertical coordinate corresponds to the total surface height, *H* (measured from $z = 0$), while the colorbar represents the film thickness, *h*. This representation allows for small variations in film thickness to be distinguished even in regions where the surface height is dominated by the underlying ramp geometry. As in the flat-substrate case, the solution remains symmetric about the midline $y=W_y/2$ because the ramp profile is independent of *y*. Climbing behavior can be characterized by tracking the front’s location along the line of symmetry. The front climbing height is defined as $H_\textrm{f}(t)=s_\textrm{r}(x_\textrm{f}^*(t))$, where $x_\textrm{f}^*(t)$ is the front position defined by the condition $h(x_\textrm{f}^*(t),W_y/2,t)=h^*$.

For relatively small forcing amplitudes (such as the case $A=1$ nm shown here), the drop quite quickly reaches a point where it can no longer climb up the ramp due to a balance between gravity, capillarity, and acoustic forcing at the advancing front. In this regime, the acoustic forcing is strongly attenuated near the front due to the increased film thickness (arising from the ramp geometry), and capillary effects dominate the subsequent dynamics. As a result, the fluid redistributes laterally and the drop spreads in the transverse (*y*) direction, as seen in Fig. 4(d).

Figure 4(e) shows a contour plot of the solution at $t=15$ s, further displaying the tendency for the drop to spread laterally when its front becomes arrested; the dashed black line indicates the start of the ramp obstacle. Figure 4(f) shows the resulting climbing height as a function of time for several forcing amplitudes. The dashed curves correspond to predictions of the 2D model, while the solid curves show the results obtained from the present 3D formulation. Since the 3D model allows transverse mass redistribution, the drop spreads laterally when the front becomes arrested, leading to a reduced asymptotic climbing height for all amplitudes when compared with the 2D predictions.

### Spreading over a bump obstacle

We next consider the spreading of an oil drop over a bump-shaped obstacle, modeled as before after an experiment, and defined in simulations by a parabolic shape profile

17 $$\begin{aligned} s_b(x) = h_o \, \max \!\left( \frac{ \sqrt{1 - \left( \dfrac{x - x_o}{w_o}\right) ^2} - \dfrac{\sqrt{3}}{2} }{ 1 - \dfrac{\sqrt{3}}{2} }, \, 0 \right) \end{aligned}$$

where we set $x_\textrm{o}=11.5$, $h_\textrm{o}=0.33$, and $w_\textrm{o}=2.8$. In contrast to the ramp considered in Sect. 4.2, the bump geometry introduces both an uphill and downhill region which produces new and interesting dynamics.

**Fig. 5:**
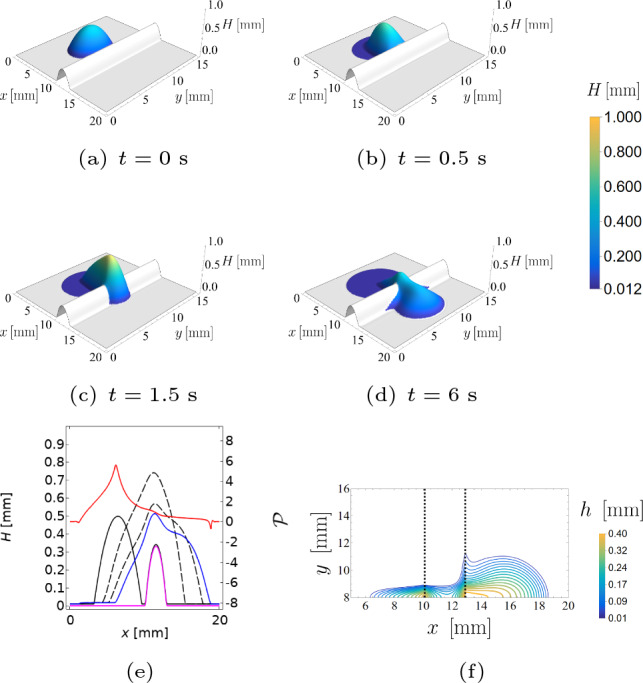
(**a**)–(**d**) Evolution of thickness profile atop bump obstacle, $H=h+s_\textrm{b}$, for $A=4$ nm. (**e**) Snapshots of the thickness profile along the midline $y=W_y/2$; the black solid line corresponds to the initial condition, the dashed black lines to the profiles at time intervals $\Delta t=2$ s, and the solid blue line to $t=6$ s. The red line shows the effective pressure, $\mathcal {P}$, at $t=6$ s, while the magenta line represents the solid obstacle. (**f**) Contour plot of the film thickness, *h*, for $A=4$ nm at $t=6$ s; the dashed black lines denote the start and end of the bump obstacle

**Fig. 6:**
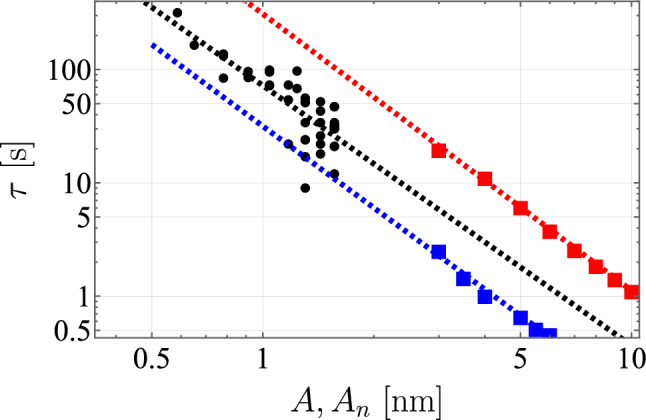
Climbing time, $\tau$, as a function of the SAW amplitude for a bump with height, $h_\textrm{o}=0.33$, and width, $w_\textrm{o}=2.8$. Experimental measurements are shown by black circles, while numerical results from the 2D and 3D models are shown by red and blue squares, respectively. The dashed lines denote power-law fits of the form, $\tau =a\bar{A}^b$, where $\bar{A}$ represents *A* for simulations and $A_n$ for experiments. The exponents are $b_\textrm{exp}=-2.29$, $b_\textrm{2D}=-2.44$, and $b_\textrm{3D}=-2.39$

Figure 5(a)–(d) shows representative snapshots of the total thickness, $H=h+s_\textrm{b}$ for $A=4$ nm. As the front encounters the obstacle, fluid accumulates on the upslope of the bump and the leading edge steepens. Continued acoustic forcing drives the drop over the crest of the obstacle, after which the bulk of the fluid spreads downstream while leaving a thin residual film behind (dark blue puddle). Figure 5(e) shows the evolution of the thickness profile along the symmetry line $y=W_y/2$. The dashed curves denote intermediate profiles while the solid blue curve shows the solution at $t=6$ s. The effective pressure distribution (red curve) reveals a strong pressure gradient near the leading edge due to the curvature of the front, which drives the front forward even when the streamwise width of the drop is large enough that the acoustic force is nearly entirely attenuated. The lateral redistribution of fluid is further illustrated by the contour plot of the thickness field shown in Fig. 5(f). One key feature evident in this plot is the lateral broadening of the drop as the bulk passes over the bump obstacle, due to capillary forces dominating during this phase of the simulation in the region of the drop where acoustic forcing is nearly fully attenuated.

A key experimentally measurable quantity for this geometry is the climbing time, $\tau$, defined as the time required for the front contact line to traverse the bump. Figure 6 shows the resulting climbing time as a function of SAW amplitude for experiments (black circles) [21], the 2D model (red squares) [21], and the 3D model (blue squares). Both simulations and experiments show a pronounced decrease in climbing time as the forcing amplitude increases. Power-law fits of the form $\tau =a\bar{A}^b$ are indicated by dashed lines in Fig. 6, showing good quantitative agreement with both formulations and a slight improvement when a fully 3D model is considered.

We note, however, that quantitative differences between experiments and simulations remain, since reproducing the experimentally observed dynamics requires larger values of the SAW amplitude in the fluid, *A*, than the experimentally measured normal displacement of the substrate, $A_n$. Similar discrepancies were also observed in the earlier 2D formulations of Fasano et al. [20]. Possible sources of these differences include the omission of inertial effects from the present leading-order long-wave model, neglected contributions from boundary-layer-driven streaming near the solid substrate, capillary waves at the free surface, and the neglect of ultrasound reflection from the free surface of the liquid. A detailed investigation of the relation between *A* and $A_n$, and of the relative importance of these effects, is left for future work.

## Conclusions

We have extended the 2D long-wave model of SAW-driven thin-film spreading to a fully 3D framework that captures transverse structure and lateral mass distribution. The model incorporates acoustic forcing through a spatially attenuated streaming profile and predicts the evolution of the film thickness, *h*(*x*, *y*, *t*). The 3D simulations show that transverse curvature and lateral flux redistribution influence the spreading dynamics, particularly near the advancing front. Specifically, the 3D formulation captures variations in the drop footprint across the transverse direction that are absent in the earlier 2D model.

Comparison with the 2D formulation reveals that the fully 3D model leads to slower fronts and lower drop apexes, due to capillary spreading in the transverse direction. In the case of a ramp obstacle, the 3D model captures transverse spreading when front propagation is arrested, in qualitative agreement with experimental observations. In the case of bump obstacles, the 3D model shows improvement (relative to the 2D model) in quantitative agreement for the power-law dependence of the climbing time on SAW amplitude.

## Supplementary Information

Below is the link to the electronic supplementary material.

Supplementary file 1 (mp4 6621 KB)

Supplementary file 2 (mp4 13983 KB)

Supplementary file 3 (mp4 14578 KB)

Supplementary file 4 (mp4 308 KB)

Supplementary file 5 (mp4 768 KB)

Supplementary file 6 (mp4 561 KB)

## Data Availability

The data that support the findings of this study are available from the corresponding author upon reasonable request.

## Computational implementation

Equation (10) is written in a form convenient for the use of COMSOL™Multiphysics PDE Coefficients Form. This package solves, by finite elements, a vectorial equation for the vector $\vec {u}=(u_1,u_2,\dots ,u_N)^\top$. The equation is of the form

18 $$\begin{aligned} \textbf{e} \frac{\partial ^2 \vec {u}}{\partial t^2}+\textbf{d} \frac{\partial \vec {u}}{\partial t} + \nabla \cdot \left( - \textbf{c} \nabla \vec {u} - \mathbf{\alpha } \, \vec {u} + \mathbf{\gamma }\right) + \mathbf{\beta } \, \nabla \vec {u} + \textbf{a} \, \vec {u} = \vec {f},\nonumber \\ \end{aligned}$$

where the coefficients of the *N* scalar equations are in the matrices $\textbf{e}$, $\textbf{d}$, $\mathbf{\gamma }$, $\textbf{a}$ (of dimensions $N\times N$), $\mathbf{\alpha }$, $\mathbf{\beta }$ (of dimensions $N\times N\times n$), $\textbf{c}$ (of dimensions $N\times N\times n \times n$) and the vector $\vec {f}$ (of dimension *N*), where *n* is the spatial dimension of the problem ($n=1,2,3$). In index notation, this equation reads as

19 $$\begin{aligned}&e_{ij}\frac{\partial ^2 u_j}{\partial t^2}+d_{ij}\frac{\partial u_j}{\partial t}+\frac{\partial }{\partial x_l} \left( -c_{ijkl} \frac{\partial u_j}{\partial x_k} -\alpha _{ijl} u_j + \gamma _{il} \right) \nonumber \\&\quad + \beta _{ijl} \frac{\partial u_j}{\partial x_l} + a_{ij} u_j = f_i, \end{aligned}$$

where $$i,j=1.\ldots ,N$$ and $$k,l=1,\ldots ,n$$.

The considered system, given by Eq. (10), contains four components $$u=(h, \mathcal {P}, \psi ^x, G)$$ ($$N=4$$) and we use four equations, namely, Eqs. (10), (13), (7), and (8) for ($$n=2)$$, corresponding to the solution depending on two spatial variables, (*x*, *y*).

In the following, we list the non-vanishing coefficients:

- Row 1 ($i=1$) for Eq. (10)
  20 $$\begin{aligned}  &   d_{11}=1,\quad c_{1211}=c_{1222}= h^3, \nonumber \\  &   \gamma _{11} =- \mathcal {C} \frac{3 \mathcal{S}}{8 K_z^4} \Psi \Big (2 K_z^2 h^2-1+ e^{2 K_z h}(1-2 K_z h) \Big )\nonumber \\ \end{aligned}$$
- Row 2 ($i=2$) for Eq. (13)
  21 $$\begin{aligned} c_{2111}= &   c_{2122} = -1, \quad a_{21}=-\textrm{Bo}, \nonumber \\  &   \quad a_{22}=1, \quad f_2=\frac{\mathcal{S} C_z}{2 K_z} \Psi \end{aligned}$$
- Row 3 ($i=3$) for Eq. (7)
  22 $$\begin{aligned} \beta _{331}=1, \quad a_{33}=2k_\textrm{s,i}H_\delta ((h+s)-h^*) \end{aligned}$$
- Row 4 ($i=4$) for Eq. (8)
  23 $$\begin{aligned} c_{4411}= &   c_{4422}=\delta _G^2, \quad a_{44}=1, \nonumber \\  &   \quad f_{4}=H_{\delta }((h+s)-h^*) \end{aligned}$$

At the domain boundaries, we apply Dirichlet boundary conditions $h= h_\textrm{p}$, where $h_\textrm{p}$ is the prewetted film thickness, and $\partial h/\partial n = 0$. It is well known (see, e.g. [24]) that the step size $\Delta \textbf{x}$ in the spatial discretization should be similar to $h_\textrm{p}$ to ensure numerical convergence, and therefore we use $\Delta x=h_\textrm{p}$ in our simulations. All reported results are obtained using $h_\textrm{p}=0.01$.
